# Translation, cultural adaptation, and validation of a smile aesthetics scale for Peruvian university students

**DOI:** 10.12688/f1000research.152728.1

**Published:** 2024-08-02

**Authors:** Evelyn Brigitte Manchego Obando, Luis Alexander Orrego-Ferreyros

**Affiliations:** 1 Facultad de Ciencias de la Salud, Escuela de Estomatología, Universidad César Vallejo, Piura, 20001, Peru

**Keywords:** Adolescent Health, Adolescent, Indicators of Quality of Life, Quality of Life, Health Related Quality Of Life

## Abstract

**Background:**

The absence of a culturally adapted and validated Smile Aesthetics Scale for Peruvian university students reveals a significant gap in aesthetic dentistry and public health research. Addressing this gap is essential for accurately assessing dental aesthetic satisfaction within this demographic, ensuring the scale’s relevance and effectiveness across Spanish-speaking cultures. The aim of the study was to translate, culturally adapt, and validate the Smile Aesthetics Satisfaction Scale for Peruvian university students.

**Methods:**

This research was applied, with an instrumental, cross-sectional, and descriptive design. Face validity was conducted with 10 students through unstructured interviews. Content validity was carried out by three experts. The validation of psychometric properties was performed on 190 students recruited through university dentistry social networks using simple random probability sampling. The Aiken test was used for face and content validity. Construct validity and internal consistency were examined through principal component and exploratory factor analysis, using promax and varimax rotations, and internal consistency was assessed with Cronbach’s Alpha.

**Results:**

Face and content validity confirmed that the items were relevant and applicable, highlighting the significance of the construct evaluated within the specific cultural context of the research. The adapted scale reflected high content validity, with a global Aiken’s V of 0.83, emphasizing the clarity, coherence, and relevance of the items according to expert evaluation. The psychometric properties of the adapted scale were exceptional among the student population, evidenced by a Cronbach’s Alpha of 0.889.

**Conclusions:**

The translation and cultural adaptation process of the Smile Aesthetics Satisfaction Scale has proven to be successful not only in terms of coherence and fidelity with the original instrument but also in its applicability and relevance within the context of university dental students.

## Introduction

Research underscores the vital role of effective public health policies in improving global quality of life and longevity, emphasizing the growing significance of university students within the young adult population. Notably, in Peru, this demographic represents a substantial portion of the youth, marking a demographic shift towards a more educated society with heightened aspirations for improved quality of life, including oral health. ^1^^,^^2^ The focus on the well-being of university students, particularly regarding their self-perception of dental aesthetics, has become increasingly prominent in public health due to its impact on self-esteem and social interactions. ^3^^–^^5^ The development of the Smile Aesthetics Satisfaction Scale for accurately evaluating smile aesthetics satisfaction underscores the necessity of its cultural adaptation and validation for use in Peru, a country with unique cultural, social, and educational characteristics. ^6^^–^^8^ While adaptations exist for diverse populations, there remains a significant research gap concerning the Peruvian university student demographic. This highlights the imperative for a linguistically and culturally pertinent version of the scale, requiring an in-depth revision of its items to ensure they resonate with the local population’s perceptions, values, and aesthetic standards. ^9^^,^^10^ The validation process ensures the scale’s reliability and validity, addressing an urgent need for precise, culturally sensitive psychometric tools to effectively measure dental aesthetics satisfaction among Peruvian university students. ^11^^–^^13^ The aim of the research was to translate, culturally adapt and validate the smile aesthetics satisfaction scale for Peruvian university students.

## Methods

### Type and design study

The study was classified as applied research with an instrumental design. Our study has adhered to the STROBE (Strengthening the Reporting of Observational Studies in Epidemiology) guidelines for cross-sectional studies. The study was approved with Official Letter from the Research Ethics Committee of the School of Stomatology N° 0119-2024-/UCV/P dated March 26, 2024. We adhered to the principles of the Declaration of Helsinki.

### Participants

The study involved 250 university stomatology students over 18 years old, registered on academic social networks, proficient in Spanish, and who consented to participate. Exclusion criteria included sensory impairments or cognitive conditions that might affect their study comprehension. Sampling varied across validation phases, utilizing three dental experts for content validity and ten students for face validity, with a sample size of 190 for psychometric validation, selected through simple random sampling. The face validity was carried out on March 30, 2024. The participants included in the study were recruited between April 6 and April 20, 2024.

### Instrument

The study utilized a survey method and structured interview technique with the Smile Aesthetics Satisfaction Scale translated, culturally adapted, and validated from its original English version proposed by Armalaite. ^14^ This instrument aims to identify the determinants of smile aesthetics as perceived by stomatology students and to examine factors that may alter the perception of smile characteristics. It comprises three sections: sociodemographic data, closed-ended questions on facial aesthetic traits, and responses to standardized photographs of 17 different human smiles. The chosen image for the questionnaire was a frontal view showing the anterior teeth, surrounding gingival tissues, and lips. This image was specifically cropped to minimize any confounding factors that could affect smile perception, ^15^ adhering to the diagnostic principles outlined by Fradeani. ^16^ According to these principles, the image must be standardized to ensure consistency across evaluations and to control for potential sources of variability. The images were taken under standardized lighting conditions using a Canon EOS Rebel T7 digital camera at a fixed distance of 50 cm from the subject, with settings adjusted to ensure uniform exposure and focus. To control for bias and variability, all photographs were taken by the same operator, and the subjects were instructed to maintain a neutral facial expression. The images were then reviewed by an independent examiner to ensure compliance with the criteria before being included in the questionnaire. Students rated each photo aesthetically using a 5-point numerical rating scale (NRS), where 1 represented the best aesthetics and 5 the worst. A score of NRS ≥ 3.5 was considered the threshold for an aesthetically unacceptable smile, as recommended by Armalaite. ^14^ This threshold was established based on previous studies that identified it as a critical point for distinguishing between acceptable and unacceptable aesthetic outcomes.

### Procedures

The study applied survey methods and structured interviews, incorporating four main validation phases: translation and cultural adaptation, face validity testing, and validation of psychometric properties, including content and construct validity, and internal consistency assessment using Cronbach’s Alpha. Analysis and validation utilized professional translation, expert review, and statistical software for reliability and clarity assessment.

### Statistical analysis

Data from surveys were first recorded and then transferred to an Excel spreadsheet for analysis with Google Sheets. A descriptive analysis including measures of central tendency for quantitative variables and frequencies for qualitative ones was conducted using JASP 0.18.3. The Aiken test, with a threshold above 0.7 and 95% confidence intervals, was employed for face and content validity, using Aiken’s V Calculator. Construct validity was assessed through principal component analysis and exploratory factor analysis, utilizing the Promax and Varimax rotation methods respectively, to identify underlying factors and ensure data simplicity. Internal consistency was evaluated with Cronbach’s Alpha, indicating good consistency with a minimum value of 0.70, using JASP 0.18.3 software.

## Results

The translation and cultural adaptation phase began with the translation of Armalaite’s study from English to Spanish by a professional translator. The translator and researcher worked together to reach a consensus, ensuring the translation’s coherence with the original. A dental surgeon with certified English skills then reviewed the translated instrument, identifying areas for improvement and confirming its linguistic and cultural suitability.

Aiken’s V coefficient analysis demonstrated high clarity (0.87 overall) and comprehension (0.86 overall) across the questionnaire’s sections among stomatology students, indicating the instrument’s strong face validity for the target population. Section 1 showed exceptional clarity with perfect scores for initial items, underscoring effective communication and understanding.

Demographic data revealed an average participant age of 23.7 years, balanced gender distribution (49.5% female, 50.5% male), and varied study years, providing a representative sample essential for interpreting the dental aesthetic satisfaction scale’s validity and applicability. Study findings emphasize the smile as universally deemed a critical aesthetic trait in social interactions, with significant gender differences in facial feature focus but no variation by academic year, underscoring the study’s relevance in evaluating smile aesthetics satisfaction among stomatology students.

The content validity of the psychometric instrument, as shown across tables, is affirmed by an overall Aiken’s V coefficient of 0.83, indicating clarity, coherence, and relevance of the scale items. Section 1 achieved perfect scores for clarity and coherence, reflecting unanimous expert agreement. Sections 2 and 3 also demonstrated high levels of clarity and relevance, with notable items indicating strong expert consensus on their significance to the construct. These results underscore the instrument’s robustness and its applicability in assessing dental aesthetic satisfaction, confirming its suitability for the research objectives and the target population’s evaluation.

The overall Measure of Sampling Adequacy (MSA) for the study was 0.824, indicating a very good fit for factor analysis, with item-specific MSAs ranging from acceptable to excellent, especially for items assessing detailed dental aesthetic perceptions. Bartlett’s Test showed a highly significant Chi-square value, supporting the suitability of data for factor analysis and highlighting the scale’s internal cohesion. These results affirm the robustness of the adapted Smile Aesthetics Satisfaction Scale and its effectiveness in assessing dental aesthetic satisfaction in the specific educational and cultural context.

Principal Component Analysis revealed a Chi-square value of 303.582, indicating an excellent fit for the model with significant inter-item relationships. The analysis identified four main components that contribute significantly to understanding dental aesthetic satisfaction among stomatology students, highlighting specific dimensions like alignment and symmetry. Promax rotation facilitated the interpretation of these components, underscoring the multidimensional nature of smile aesthetics satisfaction. Correlations between components suggest a complex interplay of perceptions, each providing unique insights into aesthetic evaluation ( Table 1).

**Table 1.** Factor-based parallel component analysis.

	RC1	RC2	RC3	RC4	Uniqueness
**D1 – I1**			0.981		0.050
**D1 – I2**			0.974		0.056
**D1 – I3**			0.930		0.114
**D2 – I1**				0.896	0.176
**D2 – I2**				0.781	0.291
**D2 – I3**				0.890	0.269
**D3 – I1**	0.710				0.472
**D3 – I2**	0.804				0.395
**D3 – I3**	0.748				0.485
**D3 – I4**	0.759				0.422
**D3 – I5**	0.612				0.481
**D3 – I6**	0.710				0.441
**D3 – I7**	0.775				0.392
**D4 – I1**		0.996			0.042
**D4 – I2**		0.878			0.110
**D4 – I3**		0.971			0.073
**D4 – I4**		0.929			0.092

Note. The rotation method is promax.

The Exploratory Factor Analysis showed a significant Chi-square value, suggesting the factorial model fits well with the empirical data, effectively capturing the underlying dimensions of dental aesthetic satisfaction among stomatology students. Varimax rotation clarified the distribution of items across four main factors, each representing specific aspects of smile aesthetics. This multidimensional structure was further elucidated through both unrotated and rotated solutions, highlighting the complexity and relevance of each dimension in assessing dental aesthetics. The analysis confirms the scale’s robustness and suitability for evaluating aesthetic satisfaction in the cultural context evaluated ( Table 2).

**Table 2.** Exploratory factor analysis: Factor loading.

	Factor 1	Factor 2	Factor 3	Factor 4	Uniqueness
**D1 – I1**			0.968		0.041
**D1 – I2**			0.955		0.061
**D1 – I3**			0.874		0.200
**D2 – I1**				0.886	0.140
**D2 – I2**				0.670	0.419
**D2 – I3**				0.686	0.478
**D3 – I1**	0.642				0.550
**D3 – I2**	0.709				0.481
**D3 – I3**	0.629				0.591
**D3 – I4**	0.687				0.498
**D3 – I5**	0.582				0.566
**D3 – I6**	0.661				0.511
**D3 – I7**	0.712				0.457
**D4 – I1**		0.920			0.032
**D4 – I2**		0.814			0.157
**D4 – I3**		0.886			0.096
**D4 – I4**		0.853			0.129

Note. The rotation method is varimax.

**Figure 1.**
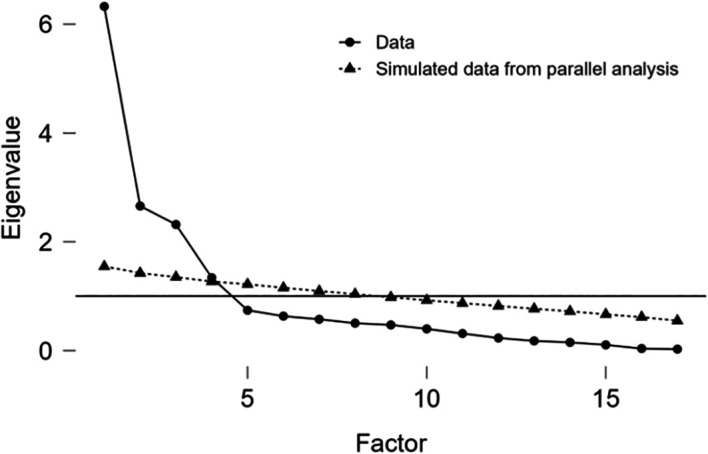
Scree plot.

The initial steep decline in the scree plot suggests a dominant first factor significantly explaining data variance, with diminishing returns from subsequent factors—indicating a strong single factor might sufficiently represent the factorial structure in stomatology student population ( Figure 1).

**Figure 2.**
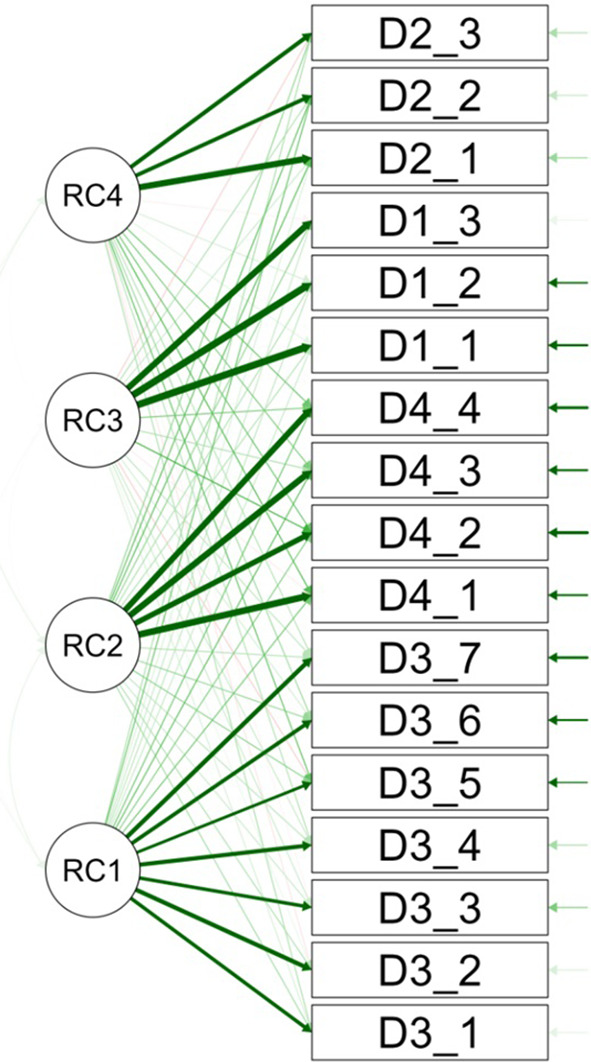
Path diagram.

The path diagram visually demonstrates the factorial structure, showing items’ associations with identified factors through line thickness, which indicates the strength of their factorial loadings, thus revealing the dimensions measured by the Smile Aesthetics Satisfaction Scale in the stomatology student population ( Figure 2).

The Smile Aesthetics Satisfaction Scale showed high internal consistency in our study, with Cronbach’s Alpha of 0.889, indicating that the scale items reliably measure a common dimension of smile aesthetic satisfaction among stomatology students in Moquegua. This level of reliability, supported by a 95% confidence interval ranging from 0.863 to 0.910, highlights the scale’s psychometric robustness and ensures its suitability for assessing dental aesthetic satisfaction accurately.

Our item reliability analysis highlighted variations in Cronbach’s Alpha upon item removal and item-total correlations, revealing that while D4 domain items significantly contribute to the scale’s overall reliability, the Smile Aesthetics Satisfaction Scale maintains high internal consistency across all items, ensuring effective capture of key dimensions in dental aesthetic satisfaction ( Table 3).

**Table 3.** Frequentist individual item reliability statistics.

Item	If item dropped	Item-rest correlation
	Cronbach’s α	
**D1 – I1**	0.714	0.417
**D1 – I2**	0.729	0.437
**D1 – I3**	0.833	0.387
**D2 – I1**	0.780	0.531
**D2 – I2**	0.870	0.591
**D2 – I3**	0.819	0.407
**D3 – I1**	0.864	0.485
**D3 – I2**	0.840	0.421
**D3 – I3**	0.864	0.389
**D3 – I4**	0.884	0.489
**D3 – I5**	0.842	0.480
**D3 – I6**	0.878	0.521
**D3 – I7**	0.891	0.512
**D4 – I1**	0.759	0.689
**D4 – I2**	0.851	0.756
**D4 – I3**	0.783	0.736
**D4 – I4**	0.917	0.753

## Discussion

The translation and cultural adaptation process of the Smile Aesthetics Satisfaction Scale in Peruvian university and cultural context has proven to be successful, maintaining coherence and fidelity with the original instrument while ensuring its applicability and relevance. The adaptation achieved conceptual precision, making the scale a reliable and understandable tool for the target population.

Face and content validity, indicated by an overall Aiken’s V of 0.83, highlighted the items’ clarity, relevance, and cultural fit. This adaptation process, reflecting a consensus on the scale’s applicability in the Peruvian environment, emphasizes the necessity of considering specific cultural characteristics in psychometric instrument adaptation. The scale’s reliability, indicated by a Cronbach’s Alpha of 0.889, significantly exceeds the acceptability threshold, affirming its utility as a measure of smile aesthetic satisfaction. ^9^^,^^17^

Exploratory factor analysis revealed a cohesive and multidimensional structure, enriching our understanding of dental aesthetic perception. This high level of translation coherence and fidelity, akin to efforts by Bela Andela S. et al., ^18^ underscores the effectiveness of collaboration between professional translators and bilingual experts. The item clarity and relevance, evaluated through face and content validity, confirmed the scale’s appropriateness and significance for university students, aligning with dental aesthetics perceptions by Pattanik S et al. ^19^

The study also noted the importance of considering individual dental dimensions variations, reflecting the cultural relevance and precision of the scale in measuring smile aesthetic satisfaction, resonating with the psychometric efficacy observed by Golshah A et al. ^20^ The exceptional internal consistency of our scale, similar to findings by Saltovic E et al., ^17^ showcases the multidimensionality and cohesion of our factorial structure, supporting the scale’s use for accurate and reliable dental aesthetic satisfaction assessment.

Our findings, alongside previous studies, underscore the importance of accurate and culturally adapted evaluation tools in dental aesthetics. The successful validation and adaptation of our scale affirms its potential as a concise alternative to longer instruments, facilitating its application in practice and research.

Despite limitations like the sample’s limited geographic scope and the homogeneity of clinical experience among participants, the study’s methodological rigor and internal consistency attest to its validity. The careful cultural adaptation of the scale, coupled with a thorough methodological implementation, highlights our work’s significance in advancing knowledge in this area. By emphasizing the need for culturally sensitive and accurate psychometric tools for dental aesthetic satisfaction assessment, our research contributes significantly to understanding dental aesthetic satisfaction in a specific context, setting a valuable precedent for future research.

The translation and cultural adaptation process of the Smile Aesthetics Satisfaction Scale was validated in Spanish not only in terms of coherence and fidelity with the original instrument but also in its applicability and relevance within the university dental student´s context.

Conclusively, this study underscores the validity and reliability of the Smile Aesthetics Satisfaction Scale as an assessment tool for dental aesthetic satisfaction. It recommends further studies to assess the scale’s applicability in broader and more diverse populations, exploring its relevance and applicability across various demographic and clinical groups. Future actions should include the scale’s implementation in clinical and educational practices and its utility in interventions aimed at enhancing aesthetic satisfaction and patients’ psychosocial well-being.

### Ethical approval and consent

The study was approved with Official Letter from the Research Ethics Committee of the School of Stomatology of Universidad César Vallejo N° 0119-2024-/UCV/P dated March 26, 2024. We adhered to the principles of the Declaration of Helsinki. Written informed consent was obtained from all participants.

## Data Availability

Figshare: Dataset smile, https://doi.org/10.6084/m9.figshare.25962646.v2. ^21^ This project contains the following underlying data:
- BD Smile scale.csv Data are available under the terms of the Creative Commons Attribution 4.0 International license (CC-BY 4.0). Figshare:Smile Aesthetics Satisfaction Scale, https://doi.org/10.6084/m9.figshare.26240450.v3. ^22^ This project contains the following extended data:
- Smile Aesthetics Satisfaction Scale.odt Data are available under the terms of the Creative Commons Attribution 4.0 International license (CC-BY 4.0).
