# Supplementary material for: Translation, cultural adaptation, and validation of a smile aesthetics scale for Peruvian stomatology students
Source: F1000Res. 2026 Mar 11;13:873. Originally published 2024 Aug 2. [Version 2] doi: 10.12688/f1000research.152728.2 (PMC13122141; doi:10.12688/f1000research.152728.2)
Supplement: Peer review history

# Reviewer report: Reviewer response for version 2

**DOI:** 10.5256/f1000research.197239.r478713

**Published:** 2026-04-27

**Authors:** Maria Conceicao Manso

**Affiliations:** 1 RISE-Health; Health Sciences Faculty, Fernando Pessoa University, Porto, Portugal

The manuscript details the translation, cultural adaptation, and psychometric validation of the "Smile Aesthetics Satisfaction Scale" for use among Peruvian stomatology (dentistry) students. This observational, cross-sectional study employed a forward-backward translation process and evaluated the instrument's face and content validity using a panel of experts and a student pilot group. Psychometric properties were assessed using data from 190 participants, utilizing Exploratory Factor Analysis (EFA), Confirmatory Factor Analysis (CFA), and Cronbach’s Alpha to determine internal consistency. The authors conclude that the scale is a reliable and culturally relevant tool for assessing dental aesthetic perception in this specific demographic.

 - The manuscript identifies a specific research gap regarding culturally sensitive tools for dental aesthetics in Peru. The revised version (v2) has successfully addressed previous concerns regarding the description of the instrument’s original conceptual structure and the four theoretical domains. The work cites relevant and contemporary literature from both global and local Peruvian contexts

 - While the core methodology (cross-sectional validation) is appropriate for the stated aims, the work lacks certain standard psychometric validation steps. Specifically, in the “REVISED Amendments from Version 1”’section, the authors acknowledge the absence of test–retest reliability and convergent validity assessments but I’m unable to find those in the actual Discussion section. Without evaluating the stability of the instrument over time or its correlation with established related measures, the technical soundness is incomplete regarding full scale validation. Therefore, authors must include a limitation regarding this aspect.

 - As another positive aspect, the manuscript provides a comprehensive description of the forward–backward translation procedure and the expert reconciliation process. The statistical methods, including the use of JASP software and specific rotation methods (Promax and Varimax), are explicitly stated. Following the STROBE guidelines further enhances the transparency of the reporting.

 - No statistical alterations are in need, as the inclusion of Confirmatory Factor Analysis (CFA) in the manuscript provides the necessary statistical rigour to support the four-factor model suggested by the EFA. The reported Cronbach’s Alpha (0.889) and Aiken’s V (0.83) are correctly interpreted as indicators of high internal consistency and content validity, respectively.

 - - The conclusions regarding the scale's applicability and reliability are well-supported by the high Aiken’s V scores for clarity and the significant factor loadings identified in the statistical analysis. The authors also correctly limit the scope of their conclusions, noting that results should not be generalised beyond stomatology students

 - Some details are, however, in need:

 Limitation: Stability Assessment - The absence of test–retest reliability is a significant limitation for a validation study. To confirm the instrument's reliability, the authors should ideally conduct a follow-up assessment with a sub-sample to calculate the Intra-class Correlation Coefficient (ICC). As you cannot include this calculation, a limitation has to be included in the discussion section

 Another limitation for the study: Validity Testing - The manuscript lacks convergent and discriminant validity. The authors should ideally compare their scale results with an established measure of oral health-related quality of life or a similar aesthetic scale to confirm it measures the intended construct.

 Reporting and Transparency (STROBE adherence):

 Study Size Justification: Following STROBE item 10, the manuscript should explicitly explain how the study size of 190 (or the initial 250) was arrived at. Was a power analysis conducted for the EFA/CFA requirements? If not, include a statement regarding it.

 Addressing Bias: Per STROBE item 9, more detail is needed on the "efforts made to address potential sources of bias". While the use of a single operator for photography is mentioned, other selection biases related to recruitment via "social networks" should be discussed.

 Generalisability: The discussion of limitations regarding the "limited geographic scope" (Moquegua) and the "homogeneity of clinical experience" is excellent. However, the authors should further elaborate on the direction and magnitude of potential biases introduced by these factors, as required by STROBE item 19.

 Minor Technical Revision:

 Abstract Consistency: The abstract mentions a sample of 190 students, while the Methods section mentions 250 students were "involved". Ensure consistent terminology between "recruited," "eligible," and "analysed" populations to satisfy STROBE item 13a.

Is the work clearly and accurately presented and does it cite the current literature?

Yes

If applicable, is the statistical analysis and its interpretation appropriate?

Yes

Are all the source data underlying the results available to ensure full reproducibility?

Yes

Is the study design appropriate and is the work technically sound?

Partly

Are the conclusions drawn adequately supported by the results?

Yes

Are sufficient details of methods and analysis provided to allow replication by others?

Yes

Reviewer Expertise:

Biostatistics, Methodology, Epidemiology

I confirm that I have read this submission and believe that I have an appropriate level of expertise to confirm that it is of an acceptable scientific standard, however I have significant reservations, as outlined above.

# Reviewer report: Reviewer response for version 1

**DOI:** 10.5256/f1000research.167524.r317951

**Published:** 2024-09-06

**Authors:** Stjepan Spalj

**Affiliations:** 1 Clinical Hospital Center Rijeka, J. J. Strossmayer University of Osijek, University of Rijeka, Kresimirova, Rijeka, Croatia

The manuscript presents results of process of validation of one instrument.

 A lot of effort is visible, and I congratulate authors, still there is a room for improvements.

 The instrument is not sufficiently described - how many dimensions does it have in original, what are the names of the dimensions? Anex contains some important information's that should be in text. If it does not have the same factor structure as original confirmatory factor analysis should be employed.

 Some steps in validation are missing. Back-translation is usual step in and comparison of original and back-translated instrument. Additionally test-retest, responsiveness, convergent and discriminant analysis.

 Sample included only dental student, but title implies that it included students of different programs. So findings can not be generalized to all students.

Is the work clearly and accurately presented and does it cite the current literature?

Partly

If applicable, is the statistical analysis and its interpretation appropriate?

Partly

Are all the source data underlying the results available to ensure full reproducibility?

Yes

Is the study design appropriate and is the work technically sound?

Partly

Are the conclusions drawn adequately supported by the results?

Yes

Are sufficient details of methods and analysis provided to allow replication by others?

Partly

Reviewer Expertise:

validation of PROMs, quality of life instruments, orthodontics

I confirm that I have read this submission and believe that I have an appropriate level of expertise to confirm that it is of an acceptable scientific standard, however I have significant reservations, as outlined above.

# Reviewer report: Reviewer response for version 1

**DOI:** 10.5256/f1000research.167524.r314555

**Published:** 2024-08-29

**Authors:** Ruchi Saini

**Affiliations:** 1 Department of Orthodontics and Dentofacial Orthopedics, Subharti Dental College, Subharti University, Meerut, Uttar Pradesh, India

The article provides a comprehensive and methodologically rigorous exploration into the cultural adaptation and validation of the Smile Aesthetics Satisfaction Scale for Peruvian university students. The research underscores the growing importance of public health policies, especially in regions like Peru, where the demographic shift towards a more educated youth is becoming increasingly prominent. The focus on university students' self-perception of dental aesthetics is timely, given its significant impact on self-esteem and social interactions.

 This article is an exemplary piece of research, offering valuable insights into the adaptation of psychometric tools for specific cultural contexts. Its meticulous approach, from the initial translation to the final validation, ensures that the Smile Aesthetics Satisfaction Scale is a reliable and culturally relevant instrument, making a significant contribution to the field of dental aesthetics and public health.

Is the work clearly and accurately presented and does it cite the current literature?

Yes

If applicable, is the statistical analysis and its interpretation appropriate?

I cannot comment. A qualified statistician is required.

Are all the source data underlying the results available to ensure full reproducibility?

Yes

Is the study design appropriate and is the work technically sound?

Yes

Are the conclusions drawn adequately supported by the results?

Yes

Are sufficient details of methods and analysis provided to allow replication by others?

Yes

Reviewer Expertise:

orthodontics, class II correctors, obstructive sleep apnea, smile aesthetics, implants, orthognathic surgeries, aligners, artificial intelligence, self ligation etc.

I confirm that I have read this submission and believe that I have an appropriate level of expertise to confirm that it is of an acceptable scientific standard.
